# Head Over Heart: An Atypical Presentation of Temporal Lobe Epilepsy

**DOI:** 10.7759/cureus.16736

**Published:** 2021-07-29

**Authors:** Laura Bradel, Ravi S Akula

**Affiliations:** 1 Internal Medicine, Arnot Ogden Medical Center, Elmira, USA; 2 Adult Cardiology, Arnot Ogden Medical Center, Elmira, USA

**Keywords:** cardio vascular disease, ictal bradycardia, sinus pauses, epilepsy disorders, electroencephalography (eeg)

## Abstract

Ictal asystole is a long-documented medical condition that causes pauses during epileptic episodes. This condition has garnered attention due to resulting accidents. The mechanism of action and treatment guidelines are not well established. We present a case of a 39-year-old male truck driver who presented with dizziness, lightheadedness, confusion, and amnesia with a history of two motor vehicle accidents within one week. During his second hospitalization he underwent pacemaker placement due to presumed sinus node dysfunction. The patient returned with recurrent symptoms and was found to have epileptic focus of the left anterior temporal lobe on electroencephalogram and was thought to have ictal asystole. In this report, we focus on the importance of evaluating for neurogenic cause of cardiac arrythmias.

## Introduction

The occurrence of asystole during epileptic episodes has been documented for many decades [1] with recent knowledge that seizures can cause cardiac arrhythmias. Ictal asystole is a transient loss of electrical activity of the heart secondary to an epileptic seizure with loss in muscle tone. It has in particular raised attention due to the concerning presenting patterns of syncope, falls, motor vehicle accidents, and fractures [2]. The mechanism of this bradyarrhythmia is not well established [3]. Four large studies reported that ictal-induced bradyarrhythmia appeared on average in less than 0.5% of patients undergoing diagnostic electroencephalogram [4-7].^ ^However, a recent small study by Rugg-Gunn et al. used loop recorders to monitor for bradyarrhythmia in patients with refractory epilepsy and found that bradyarrhythmia occurred in 15% of the patients [8]. We present a case of a patient with two recent motor vehicle accidents status post pacemaker placement for sinoatrial dysfunction who presented with recurrent symptoms of confusion and dizziness. He was diagnosed with temporal lobe epilepsy and thought to have ictal asystole.

## Case presentation

A 39-year-old-male truck driver with a past medical history of two recent motor vehicle accidents presented to the emergency department with complaints of recurrent episodes of dizziness, lightheadedness, confusion.

His first motor vehicle accident occurred while driving a truck to work. The patient was unsure if he lost consciousness at that time and felt confused after the accident. He subsequently went to the emergency department to seek further medical attention. Diagnostic studies were negative. Head computed tomography (CT) showed no acute intracranial abnormality and electrocardiogram (ECG) showed normal sinus rhythm. He was diagnosed with a concussion. There was a concern for syncope at which he underwent Holter monitoring with negative results. 

**Figure 1:**
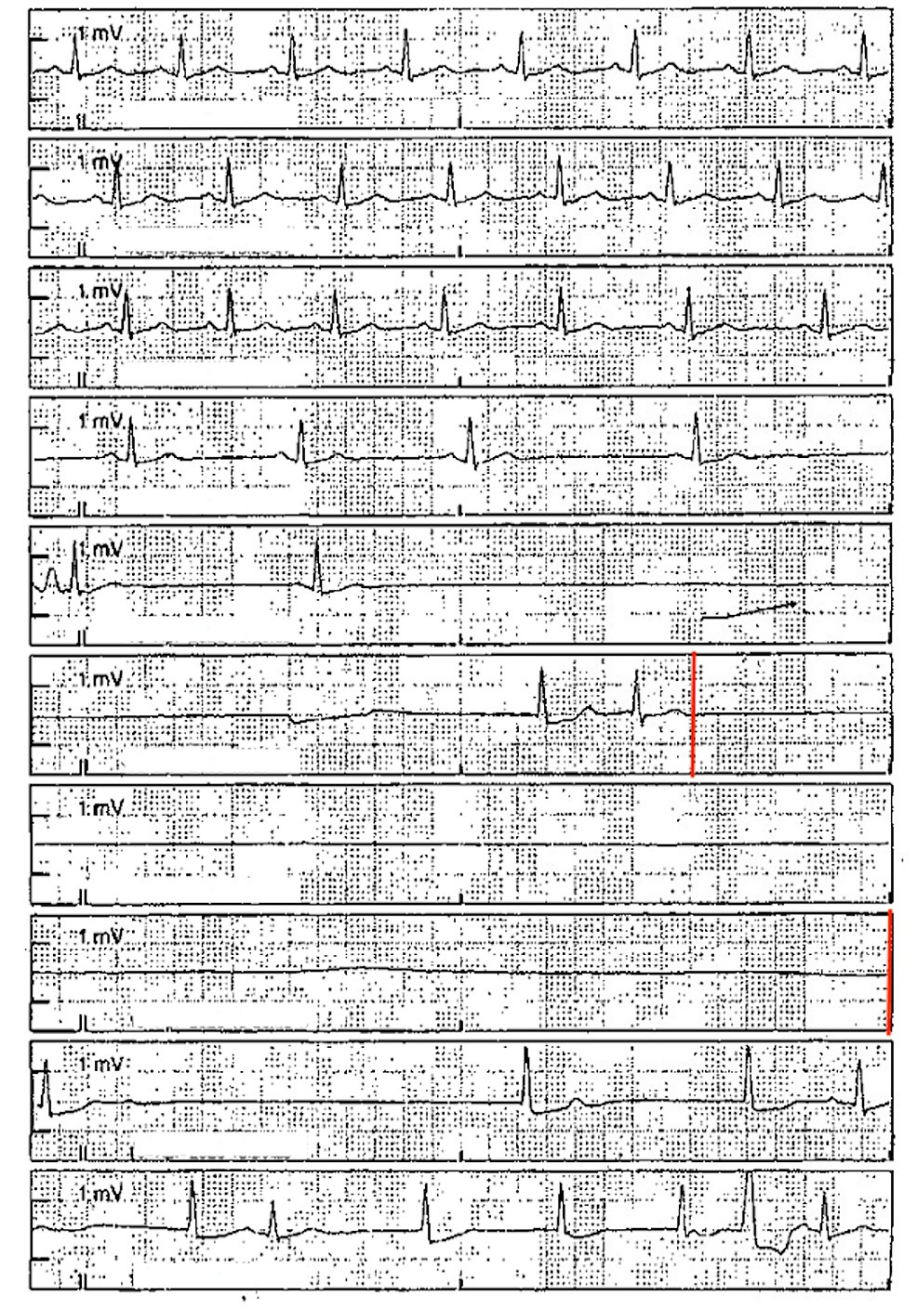
Telemetry strip showing fifteen second episode of sinoatrial asystole (red lines).

**Figure 2:**
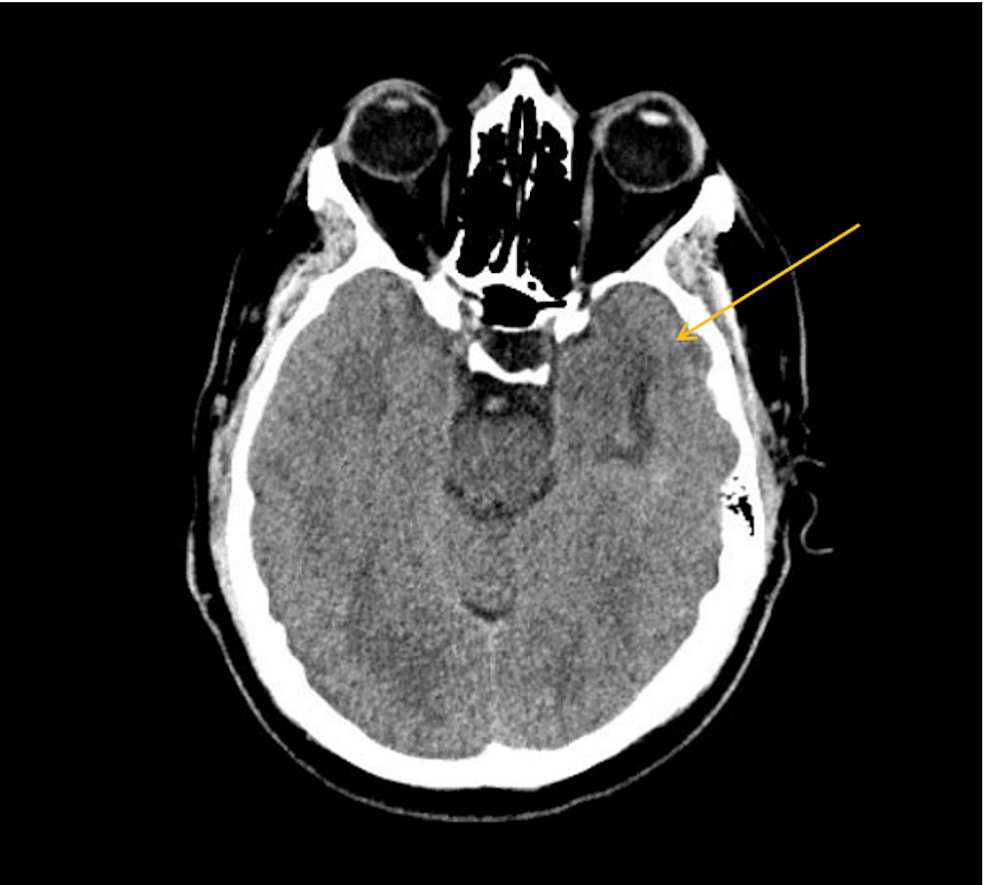
CT of the head showing chronic, small area of encephalomalacia in the left temporal lobe (yellow arrow).

**Figure 3:**
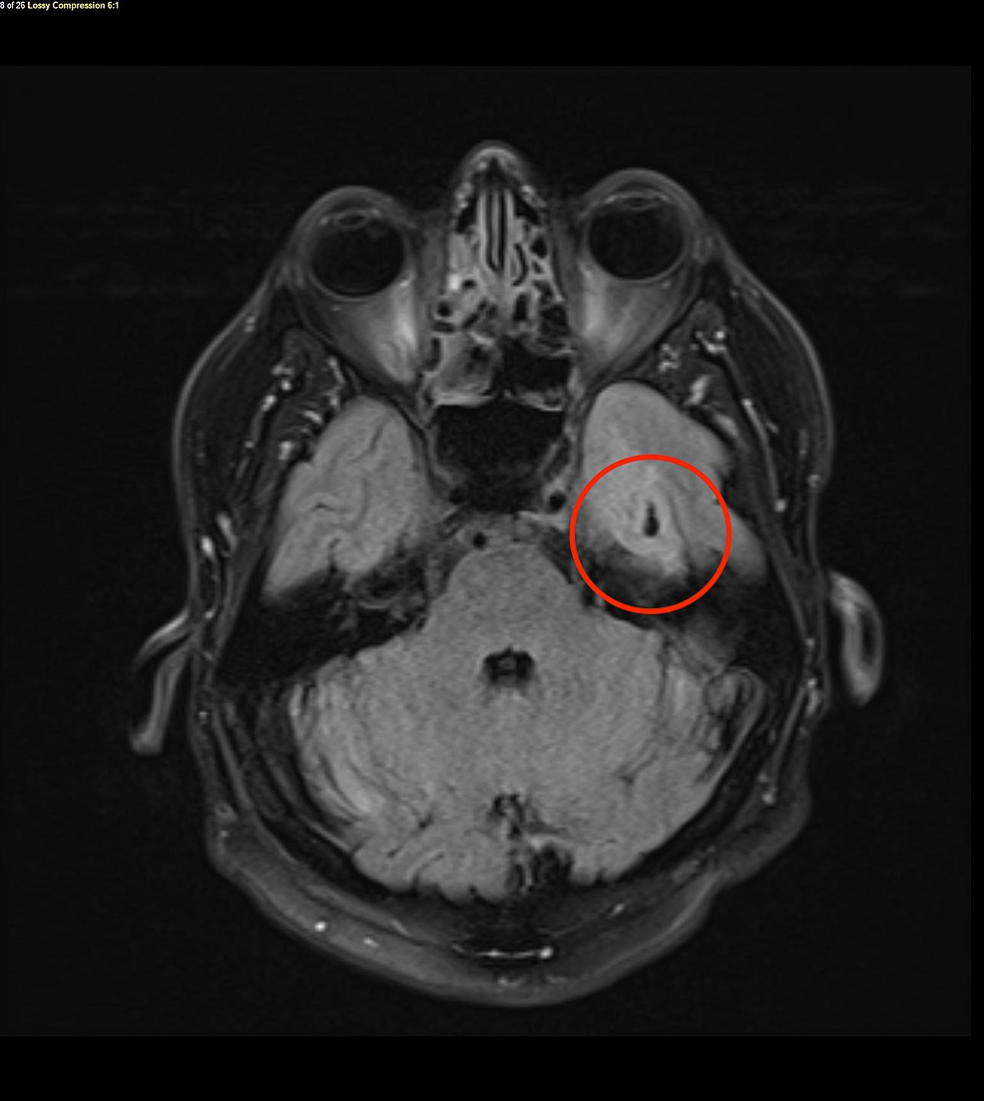
MRI of the head confirming area of left temporal encephalomalacia and gliosis (red circle).

The second event occurred one week later while driving a large truck. He was once again taken to the emergency department for further evaluation. He again reported not recalling the entire event. He was admitted for evaluation of syncope. While on telemetry he had an episode of “passing out” where he was noted to be in sinoatrial arrest lasting 15 seconds (Figure 1). An echocardiogram with bubble study showed preserved left ventricular function, no gross valvular abnormalities, or shunting. CT of the head was repeated and showed chronic encephalomalacia in the left temporal lobe which was not indentified on prior head CT (Figure 2). Later a head MRI was completed (Figure 3). The reason for the sinoatrial arrest was not entirely clear at that time. It was thought he had a syncopal episode was due to sinus node dysfunction and he underwent pacemaker placement.

**Figure 4:**
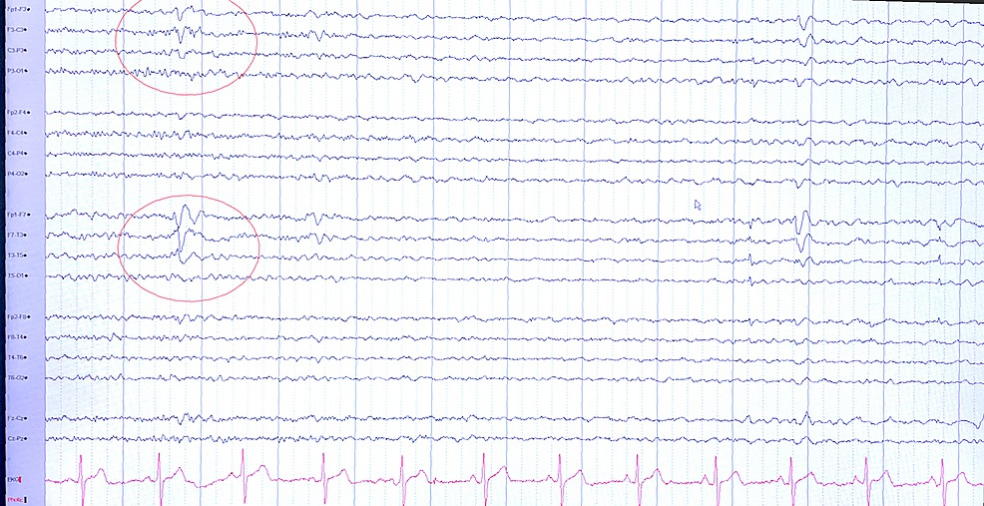
Abnormal EEG due to focal slowing and occasional spike and wave discharges over the left temporal region over the same area as the chronic small lesion found on prior head imaging in the left anterior temporal lobe (red circle).

During the third and current visit, his wife stated she witnessed her husband having periods of confusion with associated dilated pupils which lasted about an hour and a half. He once again could not recall the events. The patient denied any chest pain, dyspnea, lightheadedness, headache, leg swelling or palpitations. In the emergency department, his temperature was 97.3 degrees Fahrenheit, heart rate was 66 beats per minute, respiratory rate was 16, and blood pressure was 120/73 mmHg with oxygen saturation of 98% on room air. On physical exam, the patient was alert and oriented times three. The neurological exam was unremarkable with no focal deficits. His electrocardiogram showed normal sinus rhythm with a rate of 75, normal r-wave progression, and no acute ST-T wave changes. Potassium was 3.7mmol/l (reference range: 3.5-5.1mmol/l) and magnesium was 1.9mg/dl (reference range:1.7-2.2mg/dl). His thyroid stimulating hormone level was 3.71 lU/ml (reference range: 0.45-4.5 IU/ml). His urine drug screen was negative. High sensitivity troponins were 3 and 3pg/mL (reference range: 0-36pg/ml). The chest x-ray showed no acute cardiopulmonary process. His pacemaker was interrogated which showed no events of arrhythmia. Repeat echocardiogram showed preserved ejection fraction of 50-55% with normal valvular function, and no structural disease. This time, there was concern for seizure activity and was admitted for further evaluation. He underwent repeat head CT which was unchanged from prior. Electroencephalogram (EEG) monitoring showed epileptic episodes with focus on the left anterior temporal area over the same area as noted on the head CT (Figure 4). It was thought the area of encephalomalacia was inciting seizure activity. He was started on carbamazepine 200 mg twice a day for seizure prophylaxis. 

Upon follow up with neurology and cardiology, the initial concussion during the first motor vehicle accident irritated a small focus of encephalomalacia in the left temporal lobe that was not spotted on initial head CT inciting simple and partial complex seizures. It was thought at the time that the episode of asystole was from sinus node dysfunction and the patient underwent pacemaker placement, however he continued to have recurrent symptoms of amnesia and dizziness. It was later identified at repeat hospitalization that he was having seizures that likely caused the prior ictal asystole. He was advised to no longer operate heavy machinery. Since the motor vehicle accidents, the patient reported memory problems, attention lapses, muscle twitches, and spasms.

## Discussion

The diagnosis of ictal asystole can be challenging unless it is suspected. It can be captured on EEG monitoring or long-term monitoring with implantable loop recorder. History taking is essential with special attention to seizure like activity with of history of traumatic falls and accidents [7,9].

The exact mechanism of ictal asystole is not yet fully understood, however multiple mechanisms have been proposed. Ictal asystole has a strong association with temporal lobe epilepsy, most often the trigger is a complex partial seizure [10]. One likely mechanism is that a seizure onset in the left hemisphere results in a direct effect leading to bradycardia [3] as the right vagal nerve innervates the sino-atrial node [7]. Another mechanism is that ictal asystole is caused indirectly by the seizure through catecholamine release, evoking a vasovagal reflex [11].

Management of ictal induced bradyarrhythmia is primarily through use of antiepileptics or surgery to prevent seizure episodes. There are currently no guidelines on indications of cardiac pacing in this subset for prevention of ictal-induced bradycardia. It is suggested that refractory symptomatic bradycardia despite antiepileptics and surgery or pauses ≥6 seconds could be used as a threshold to consider pacing [7,12].

## Conclusions

With limited data on cardiac arrhythmias during epileptic seizures, it is important to consider neurogenic cause during evaluation. Careful history taking, telemetry, and EEG monitoring become essential in these situations.
